# A Novel Algebraic Saturation-Based PID Controller Optimized by Animated Oat Algorithm for Ultra-Fast Dynamic Response of Automatic Voltage Regulation

**DOI:** 10.3390/biomimetics11050343

**Published:** 2026-05-14

**Authors:** Ömer Türksoy

**Affiliations:** Department of Electrical and Electronics Engineering, Iskenderun Technical University, 31200 İskenderun, Hatay, Turkey; omer.turksoy@iste.edu.tr

**Keywords:** automatic voltage regulator, algebraic saturation-based PID controller, metaheuristic optimization, AOOA, nonlinear control

## Abstract

This paper presents a novel algebraic saturation-based Proportional–Integral–Derivative (ASB-PID) controller for achieving ultra-fast and well-damped dynamic response in automatic voltage regulator (AVR) systems. The proposed controller incorporates an algebraic saturation-based nonlinear transformation applied to both the error signal and its derivative, enabling adaptive control sensitivity across different operating regions. This formulation preserves high sensitivity near the equilibrium point while effectively limiting excessive control action under large transient deviations, thereby overcoming the inherent trade-off between response speed and overshoot observed in conventional PID-based controllers. To address the highly nonlinear and multimodal tuning problem, the controller parameters are optimally determined using the Animated Oat Optimization Algorithm (AOOA), which provides strong global exploration capability and stable convergence behavior. The effectiveness of AOOA is first validated through comparative analysis with widely used metaheuristic algorithms, including Particle Swarm Optimization (PSO), Gray Wolf Optimizer (GWO), Whale Optimization Algorithm (WOA), and Sine Cosine Algorithm (SCA). Furthermore, the proposed controller is benchmarked against recently developed high-performance AVR control strategies, including Gudermannian-PID (G-PID), fractional-order PID (FOPID), and higher-order PID-based controllers. Simulation results demonstrate that the proposed AOOA-optimized ASB-PID controller achieves a rise time of 0.0215 s and a settling time of 0.0383 s with zero overshoot and negligible steady-state error, significantly outperforming both competing optimization algorithms and state-of-the-art control designs. Comprehensive benchmarking further confirms that the proposed method consistently delivers superior performance in terms of speed, stability, and robustness, indicating that it provides an effective, computationally efficient, and scalable solution for high-performance AVR systems and broader nonlinear control applications. Unlike conventional nonlinear PID designs based on hyperbolic or sigmoid mappings, the proposed algebraic formulation provides a more explicit and effective saturation mechanism, enabling a superior balance between transient speed and overshoot suppression without increasing controller complexity.

## 1. Introduction

### 1.1. Background on AVR Challenges in Modern Power Systems

The increasing prevalence of renewable energy sources and power electronic interfaces has significantly altered the dynamic behavior of modern power systems. Consequently, voltage regulation is no longer a simple task managed by relatively slow electromechanical dynamics, but rather a fast and highly nonlinear control problem subject to uncertainties, disturbances, and operating condition variations. In modern power grids, rapid load fluctuations, intermittent renewable generation, and converter-dominated dynamics introduce additional complexity, making the voltage regulation task more challenging than in conventional systems.

In this context, the AVR plays a critical role in maintaining the terminal voltage of synchronous generators within acceptable limits. However, achieving both fast transient response and well-damped steady-state behavior simultaneously remains a challenging goal, especially under rapidly changing system conditions [1,2,3,4,5]. In particular, the presence of system nonlinearities, parameter uncertainties, and external disturbances makes it difficult to design a controller that can consistently deliver high performance across different operating regions.

### 1.2. Conventional and Advanced PID Controllers

Conventional PID controllers have been the most common solution in AVR systems for many years due to their structural simplicity and ease of implementation. However, classical PID controllers, usually formulated as single-degree-of-freedom (1DOF) structures, are limited in their ability to adequately represent the nonlinear and time-varying dynamics of modern power systems [6]. In particular, the intrinsic coupling between reference tracking and disturbance suppression presents a fundamental trade-off problem between fast response and overshoot suppression. This trade-off problem becomes even more pronounced in operating conditions with parameter uncertainties and fast disturbances; in such cases, it is often not possible to simultaneously achieve fast dynamic behavior, sufficient damping, and low overshoot with a fixed set of controller parameters. Therefore, classical PID controllers may fail to exhibit consistent performance across different operating regions, necessitating the development of more flexible and high-performance control structures for AVR applications [7,8].

To overcome the limitations of classical PID controllers, various enhanced PID-based structures have been proposed in the literature to improve flexibility and transient performance. One of the most prominent approaches is the two-degree-of-freedom (2DOF) PID controller, which separates reference tracking and disturbance rejection, thereby enabling more effective shaping of the transient response [9,10,11]. In addition to 2DOF configurations, higher-order extensions such as proportional–integral–derivative–acceleration (PIDA) controllers have been introduced to better capture system dynamics by incorporating additional dynamic terms [8,12,13]. Similarly, advanced variants including PIDD^2^, RPIDD^2^, and RPIDD^2^-PI controllers employ multiple derivative components to enhance damping characteristics and suppress oscillatory behavior [6,14,15]. Although these approaches provide noticeable improvements in transient performance, they generally increase the structural complexity of the controller and introduce a larger number of tuning parameters, which may limit their practical applicability, particularly in real-time AVR implementations.

### 1.3. Fractional-Order and Intelligent Control Strategies

In parallel, fractional-order control strategies have attracted significant attention due to their ability to introduce additional degrees of freedom in controller design. FOPID controllers, defined by non-integer integration and differentiation orders, provide enhanced flexibility in shaping system dynamics and improving robustness under nonlinear operating conditions [16,17,18,19,20]. Several studies have shown that hybrid structures such as FOPIDD^2^ and fractional Proportional–Integral–Derivative with Filter (PIDF) controllers can achieve improved transient and steady-state performance in AVR systems when properly tuned [21,22,23,24,25]. However, the expanded parameter space makes the tuning process more challenging and may limit their applicability in time-sensitive implementations.

Beyond fractional-order approaches, hybrid and intelligent control strategies have also been widely investigated. Fuzzy logic-based PID and PIDF controllers enable adaptive behavior through rule-based mechanisms, allowing improved handling of system nonlinearities and uncertainties [26,27,28]. In addition, cascaded and multi-loop control structures have been proposed to enhance dynamic performance by separating control objectives and enhancing stability margins [29,30]. Despite their effectiveness, these approaches often rely on multiple design layers or heuristic rules, which may complicate parameter selection and practical implementation.

In addition to hybrid approaches, sliding mode control (SMC) has also been extensively applied to AVR systems due to its strong robustness against uncertainties and disturbances. Advanced SMC-based designs have demonstrated fast convergence and effective disturbance rejection under nonlinear conditions [31,32,33]. However, practical implementation of SMC methods is often limited by issues such as chattering and the need for careful design of the sliding surface, which may adversely affect overall system performance [34].

In recent years, an alternative approach has emerged in the form of nonlinear function-based controllers. These controllers aim to enhance system performance by embedding nonlinear mappings directly into the control structure, thereby improving adaptability without significantly increasing complexity. Various nonlinear functions, including sigmoid, hyperbolic, and trigonometric mappings, have been utilized to design adaptive control laws [35,36,37,38]. Such approaches allow the controller to adjust its sensitivity according to the magnitude of the error signal, leading to improved transient response and reduced overshoot. However, existing function-based controllers are generally limited to smooth nonlinear transformations, which may not provide sufficiently strong saturation characteristics under large transient deviations [39]. In particular, achieving both high sensitivity near the equilibrium region and effective attenuation under large error conditions remains a challenging problem.

### 1.4. Research Gaps and Objectives

Despite the extensive research on AVR controller design, achieving a consistent and well-balanced dynamic performance remains a challenging problem. In particular, existing control approaches still struggle to simultaneously ensure fast transient response, low overshoot, and robust behavior under varying operating conditions [7,8,9,16,17,18,19,20].

Moreover, current control strategies exhibit several inherent limitations. Classical PID controllers are constrained by a fundamental trade-off between response speed and overshoot suppression, which restricts their performance in practical applications [6,7,8]. Although advanced and higher-order PID-based structures improve transient characteristics, they introduce increased structural complexity and a larger number of tuning parameters, which may reduce their practical applicability [12,13,14,15]. Similarly, fractional-order and intelligent control strategies provide enhanced flexibility and robustness; however, this comes at the cost of an expanded parameter space and increased computational burden, making the tuning process more challenging [16,17,18,19,20,21,22,23,24,25,26,27,28,29,30].

Furthermore, nonlinear function-based controllers often rely on smooth nonlinear mappings that may not provide sufficiently strong saturation under large transient deviations [35,36,37,38]. As a result, achieving an effective balance between high sensitivity near the equilibrium region and strong attenuation during large disturbances remains an open problem in AVR control design [39].

In addition to controller design, parameter tuning represents a critical challenge in AVR systems due to the inherently nonlinear, multimodal, and high-dimensional nature of the optimization problem [40,41,42]. Although a wide range of metaheuristic algorithms, including PSO, GWO, WOA, and SCA, have demonstrated promising performance, they may still suffer from limitations such as premature convergence and an imbalance between exploration and exploitation capabilities [43,44,45,46,47,48,49,50,51,52]. Therefore, the selection of an appropriate optimization algorithm plays a crucial role in achieving reliable and high-quality controller tuning.

Motivated by these limitations, the main objective of this study is to propose a novel algebraic saturation function-based PID controller for AVR systems. In the proposed structure, nonlinear transformations are applied to both the error signal and its derivative to enable adaptive sensitivity across different operating regions. Unlike conventional smooth nonlinear mappings, the proposed algebraic formulation provides stronger and more explicitly controllable saturation characteristics. This enables rapid response under large transient deviations while maintaining smooth and precise behavior near the equilibrium point. As a result, an effective balance between fast dynamic performance and reduced oscillatory behavior is achieved without significantly increasing controller complexity.

For parameter tuning, the Animated Oat Optimization Algorithm (AOOA) is employed due to its strong global exploration capability and effective convergence characteristics [53]. Inspired by adaptive behavioral mechanisms observed in natural systems, AOOA belongs to the class of biomimetic and bio-inspired optimization algorithms that emulate dynamic interaction and adaptive search processes to effectively balance exploration and exploitation during optimization. In recent years, such nature-inspired optimization strategies have attracted considerable attention in modern control engineering because of their robustness and flexibility in solving nonlinear and multimodal optimization problems. In AVR applications, where controller tuning involves highly coupled and nonlinear dynamic characteristics, biomimetic optimization approaches provide an effective framework for achieving reliable and high-quality parameter tuning performance.

The effectiveness of the proposed approach is validated through a comprehensive evaluation framework. The controller parameters are optimized using AOOA and compared with widely used metaheuristic algorithms, including PSO, GWO, WOA, and SCA. In addition, the proposed controller is benchmarked against recent high-performance AVR control methods, such as the Starfish Optimization Algorithm (SFOA)-optimized G-PID [36], Superb Fairy-Wren Optimization Algorithm (SFWOA)-optimized PID [48], Seagull Optimization Algorithm (SOA)-optimized FOPID [17], Fireworks Whale Optimization Algorithm (FWWOA)-optimized PIDD^2^ [14], and Non-Monopolize Optimization (NO)-optimized PIDA [13] controllers. The results demonstrate that the proposed method achieves a favorable trade-off between fast transient response, reduced overshoot, and improved damping while preserving structural simplicity and practical applicability.

The main contributions of this study can be summarized as follows:

• A novel algebraic saturation function-based controller is proposed for AVR systems, introducing an effective nonlinear mapping applied to both the error signal and its derivative to enhance dynamic response characteristics.
• The proposed controller achieves improved transient performance, including fast response, reduced overshoot, and enhanced damping, while maintaining a simple and implementable structure.
• The AOOA is employed for controller parameter tuning, and its effectiveness is demonstrated through comparative analysis with widely used metaheuristic algorithms, including PSO, GWO, WOA, and SCA.
• A comprehensive two-level comparative framework is established, in which both the optimization algorithm and the controller structure are systematically evaluated.
• The proposed controller is further validated against recent state-of-the-art AVR control strategies, including SFOA-optimized G-PID, FWWOA-optimized PIDD^2^, NO-optimized PIDA, SFWOA-optimized PID and SOA-optimized FOPID controllers, demonstrating its effectiveness and competitiveness.
• The proposed approach provides an effective balance between performance improvement and structural simplicity, making it suitable for practical implementation in modern power systems.

## 2. Description of the AVR System

The AVR system considered in this study is a widely used benchmark model in power system control studies due to its capability to represent the essential dynamics of synchronous generator excitation systems. The AVR plays a critical role in maintaining the terminal voltage at a desired reference level by regulating the excitation voltage of the generator.

**Figure 1:**
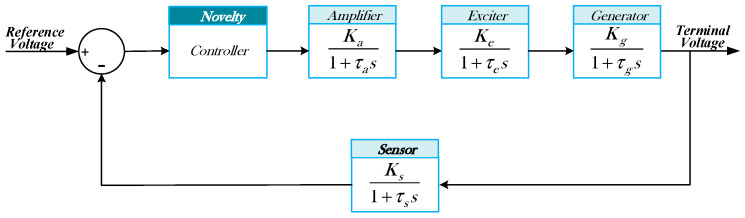
Block diagram of the traditional AVR system.

The AVR system is typically composed of four main components: the amplifier, exciter, generator, and sensor. Each component can be approximated by a first-order transfer function, which provides a sufficiently accurate representation of the system dynamics for controller design and performance analysis [54,55]. The overall block diagram of the AVR system is illustrated in Figure 1.

The transfer functions of the AVR components are given as follows:

• Amplifier:

(1) $$G_{A}\left(s\right)\;=\;\frac{K_{A}}{1+\tau_{A}s}$$

• Exciter:

(2) $$G_{E}\left(s\right)\;=\;\frac{K_{E}}{1+\tau_{E}s}$$

• Generator:

(3) $$G_{G}\left(s\right)\;=\;\frac{K_{G}}{1+\tau_{G}s}$$

• Sensor:

(4) $$G_{S}\left(s\right)\;=\;\frac{K_{S}}{1+\tau_{S}s}$$

As shown in Figure 1, these components are connected in cascade to form the forward path of the system, while the sensor is placed in the feedback loop. The reference voltage is compared with the measured terminal voltage to generate the error signal, which serves as the input to the controller. The controller output regulates the excitation system, thereby controlling the generator terminal voltage.

The ranges and selected values of the AVR parameters used in this study are summarized in Table 1. These values are chosen based on commonly adopted benchmark settings in the literature to ensure a fair and consistent performance evaluation [32,54,55,56,57,58]. Although the amplifier and sensor time constants are located at the lower bounds of their reported ranges, they represent a fast-response nominal AVR configuration commonly used for controller performance evaluation. To avoid bias toward a single operating point, robustness analyses are further conducted in Section 5.3 by varying the main AVR time constants over a wide range, confirming that the proposed controller remains stable and effective under significant parameter uncertainties.

## 3. Proposed Algebraic Saturation-Based PID Controller

The control objective in AVR systems is to achieve fast voltage tracking with minimal overshoot and well-damped dynamic behavior under varying operating conditions. However, as discussed in the previous section, conventional and advanced control strategies often fail to simultaneously satisfy these requirements due to inherent trade-offs between response speed, damping characteristics, and robustness.

To overcome these limitations, a novel ASB-PID controller is proposed in this study. The proposed controller modifies the classical PID structure by introducing nonlinear transformations applied to both the error signal and its derivative, thereby enabling adaptive sensitivity across different operating regions.

The voltage tracking error is defined as

(5) $$e\left(t\right)\;=\;V_{ref}\left(t\right)-V_{t}\left(t\right)$$

where *V_ref_*(*t*) is the reference voltage and *V_t_*(*t*) is the terminal voltage of the generator. In the proposed structure, the error signal and its derivative are first scaled as

(6) $$\begin{array}{ccc} x_{e}\left(t\right)\;=\;\beta_{e}e\left(t\right) & , & x_{d}\left(t\right)\;=\;\beta_{d}\dot{e}\left(t\right) \end{array}$$

where *β_e_* and *β_d_* are scaling parameters. The scaling parameters *β_e_* and *β_d_* are introduced to adjust the magnitude of the error signal and its derivative before applying the nonlinear saturation function. This scaling step ensures that the input signals are appropriately positioned within the effective operating region of the algebraic saturation function. If the input signals are too small, the nonlinear transformation may behave almost linearly, reducing its impact. Conversely, excessively large inputs may lead to premature saturation and overly aggressive control limitation.

Therefore, *β_e_* and *β_d_* act as input conditioning parameters that normalize the signals and enhance the effectiveness of the subsequent nonlinear mapping. This role is fundamentally different from that of the saturation parameters *μ_e_* and *μ_d_*, which directly control the shape and strength of the nonlinear saturation. By decoupling input scaling and saturation behavior, the proposed controller provides greater flexibility and improved control performance.

The core component of the proposed controller is the algebraic saturation function, defined as

(7) $$\vartheta \left(x\right)\;=\;\frac{x}{\sqrt{1+\mu x^{2}}}$$

where *μ* > 0 is a design parameter that determines the saturation level.

To further clarify the advantage of the proposed algebraic saturation function, it is instructive to compare it with commonly used nonlinear mappings such as the hyperbolic tangent [37,38], sigmoid [35], and Gudermannian [36] functions. These functions are widely employed in nonlinear PID designs due to their smoothness and bounded output characteristics. However, they exhibit relatively gradual saturation behavior, which may limit their effectiveness in suppressing excessive control action during large transient deviations.

In contrast, the proposed algebraic saturation function provides a more aggressive yet smoothly controlled saturation mechanism. Specifically, for large values of |*x*|, the function asymptotically behaves as $\vartheta (x)\approx \sqrt{\frac{sign(x)}{\sqrt{\mu }}}$, leading to faster attenuation of the control signal compared to tanh or sigmoid-based mappings. At the same time, around the origin, the function retains an approximately linear characteristic (ϑ(x)≈x), ensuring high sensitivity and precise regulation in the steady-state region.

Another important distinction lies in the explicit tunability of the saturation strength through the parameter *μ*. Unlike hyperbolic, sigmoid and Gudermannian functions, where the saturation behavior is indirectly controlled, the proposed formulation allows direct adjustment of the trade-off between sensitivity and saturation. This property is particularly advantageous for AVR systems, where both rapid transient suppression and accurate steady-state tracking are required.

Therefore, the proposed algebraic saturation function provides a more effective balance between fast response, reduced overshoot, and smooth control action compared to conventional nonlinear mappings, while maintaining a simple and computationally efficient structure.

**Figure 2:**
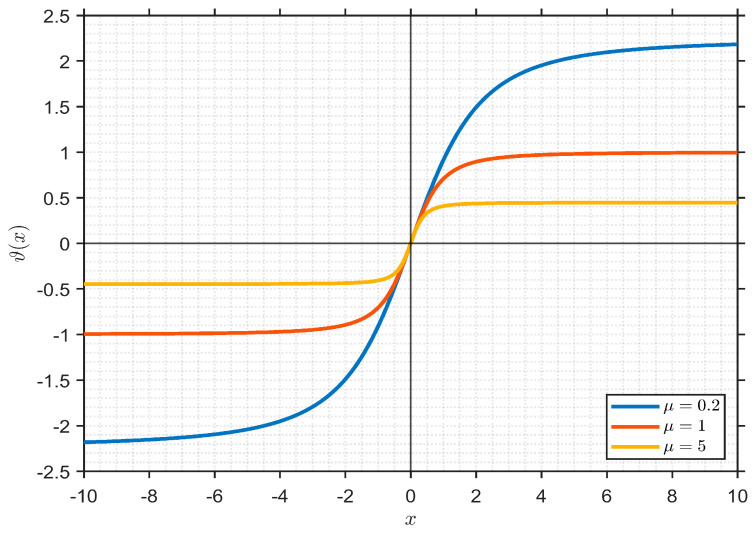
Effect of the parameter *μ* on the input–output characteristic of the proposed algebraic saturation function.

The input–output characteristic of the algebraic saturation function for different values of *μ* is illustrated in Figure 2.

As shown in the figure, the parameter *μ* directly affects the saturation level of the function while preserving its smooth and symmetric behavior. Smaller values of *μ* result in a wider linear region and weaker saturation, whereas larger values lead to stronger saturation and earlier limiting of the control signal. This tunable characteristic provides additional flexibility in shaping the controller response according to system requirements.

From a control design perspective, the parameter *μ* can be interpreted as a saturation-strength coefficient that should be selected according to the expected operating range and nonlinearity level of the AVR system. For systems operating predominantly around the equilibrium point with relatively small voltage deviations, smaller values of *μ* are preferable, as they preserve a wider quasi-linear region and ensure high control sensitivity. In contrast, for systems subject to large disturbances, parameter variations, or significant transient deviations, larger values of *μ* provide stronger saturation, effectively limiting excessive control action and reducing overshoot.

In the proposed ASB-PID structure, separate parameters *μ_e_* and *μ_d_* are used for the error signal and its derivative, respectively, allowing independent shaping of the nonlinear response. To avoid empirical tuning and to ensure optimal performance, these parameters are not manually selected; instead, they are included in the optimization vector and automatically tuned by the AOOA within predefined feasible bounds. This approach enables the controller to adapt its nonlinear characteristics according to the closed-loop AVR dynamics and ensures a balanced trade-off between responsiveness and stability.

From a robustness perspective, moderate deviations in the saturation parameters do not significantly degrade the overall control performance. This is mainly due to the smooth and bounded nature of the algebraic saturation function, which prevents abrupt variations in the control signal. As a result, the proposed controller maintains stable and well-damped behavior even in the presence of saturation parameter mismatch.

Unlike conventional saturation functions or discontinuous nonlinear mappings, the proposed algebraic formulation is smooth and continuously differentiable over the entire domain. This property helps to avoid undesirable effects such as abrupt control actions or chattering. Furthermore, the symmetric structure of the function ensures consistent behavior for both positive and negative input signals, which is essential for bidirectional error dynamics in AVR systems.

The algebraic saturation transformation is applied separately to the scaled error and its derivative:

(8) $$\begin{array}{cc} z_{e}\left(t\right)\;=\;\frac{x_{e}\left(t\right)}{\sqrt{1+\mu_{e}x_{e}^{2}\left(t\right)}} & \begin{array}{cc} , & z_{d}\left(t\right)\;=\;\frac{x_{d}\left(t\right)}{\sqrt{1+\mu_{d}x_{d}^{2}\left(t\right)}} \end{array} \end{array}$$

where *μ_e_* and *μ_d_* are design parameters controlling the saturation levels.

These signals are then combined to form an intermediate control signal:

(9) $$\eta \left(t\right)\;=\;\omega_{e}z_{e}\left(t\right)+\omega_{d}z_{d}\left(t\right)$$

where *w_e_* and *w_d_* are weighting coefficients. The weighting factors *w_e_* and *w_d_* determine the relative contribution of the nonlinear error and derivative components in forming the intermediate control signal *η*(*t*). Unlike the scaling parameters *β_e_* and *β_d_*, which are applied before the nonlinear transformation to condition the input signals, the weighting factors are applied after the algebraic saturation function and directly influence the composition of the control signal.

From a control perspective, *w_e_* emphasizes the contribution of the nonlinear error component, which primarily affects steady-state accuracy and overall tracking performance, while *w_d_* adjusts the influence of the nonlinear derivative component, which is mainly associated with damping characteristics and transient response shaping. Therefore, these parameters provide an additional degree of freedom in tuning the balance between responsiveness and stability.

By separating input scaling (*β_e_*, *β_d_*), nonlinear shaping (*μ_e_*, *μ_d_*), and post-transformation weighting $\left(\omega_{e},\omega_{d}\right)$, the proposed ASB-PID controller offers a structured and flexible design framework, allowing independent adjustment of different aspects of the control behavior.

Finally, the control input is generated using a PID structure driven by *η*(*t*):

(10) $$u\left(t\right)\;=\;K_{p}\eta \left(t\right)+K_{i}\int \eta \left(\tau \right)+K_{d}\eta_{f}\left(t\right)$$

where *K_p_*, *K_i_* and *K_d_* are the proportional, integral, and derivative gains, respectively. The filtered derivative component *ηf*(*t*) is defined in the Laplace domain as

(11) $$\eta_{f}\left(s\right)\;=\;\frac{\omega_{f}s}{s+\omega_{f}}\eta \left(s\right)$$

where *w_f_* denotes the derivative filter coefficient. This filtering stage is introduced to attenuate high-frequency noise and to provide a smoother derivative action in the controller output.

**Figure 3:**
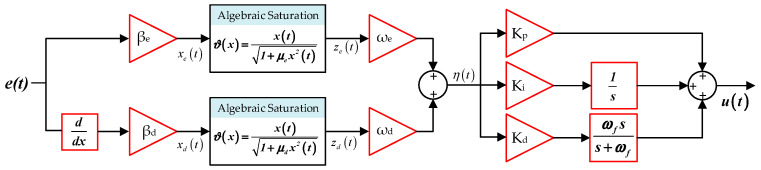
Block diagram of the proposed ASB-PID controller.

The overall structure of the proposed ASB-PID controller is illustrated in Figure 3.

As shown in the figure, the error signal and its derivative are first processed through scaling blocks and algebraic saturation functions to generate the nonlinear signals *z_e_*(*t*) and *z_d_*(*t*). These signals are then weighted and combined to form the intermediate control signal *η*(*t*), which is subsequently fed into the PID structure. The proportional, integral, and derivative actions are applied to *η*(*t*), and the final control signal is obtained by combining these components. This representation clearly demonstrates how the proposed controller integrates nonlinear shaping with a classical PID framework in a unified architecture.

The proposed ASB-PID controller combines the advantages of linear PID control and nonlinear saturation-based shaping. The algebraic transformation ensures high sensitivity in the vicinity of the equilibrium point, enabling precise voltage regulation, while effectively limiting excessive control action during large transient deviations. As a result, the controller achieves fast dynamic response with reduced overshoot and improved damping characteristics, without introducing significant structural complexity.

Compared to trigonometric or hyperbolic nonlinear mappings commonly used in the literature, the proposed algebraic formulation offers a simpler structure with explicitly controllable saturation behavior. This makes the ASB-PID controller particularly suitable for real-time AVR applications, where both computational efficiency and control performance are critical.

From a frequency-domain perspective, the proposed nonlinear controller can be locally interpreted through linearization around the operating point. In this region, the algebraic saturation function behaves approximately linearly, and the controller exhibits characteristics similar to a classical PID structure. Therefore, the stability and damping properties can be understood in terms of equivalent linear dynamics, while the nonlinear saturation enhances robustness under large disturbances.

## 4. Optimization of the ASB-PID Controller Using AOOA

### 4.1. Optimization Problem Formulation

The performance of the proposed ASB-PID controller depends on the appropriate selection of its parameters. Due to the nonlinear transformations introduced by the algebraic saturation functions and the strong coupling among the controller parameters, the tuning problem becomes nonlinear and multimodal. Therefore, the controller tuning task is formulated as an optimization problem in which the objective is to determine the optimal parameter vector that minimizes a performance index defined based on the AVR system response.

For the proposed ASB-PID controller, the parameter vector is defined as

(12) $$\Theta \;=\;[K_{p},K_{i},K_{d},\beta_{e},\beta_{d},\mu_{e},\mu_{d},\omega_{e},\omega_{d},\omega_{f}]$$

where *K_p_*, *K_i_* and *K_d_* are the PID gains, *β_e_* and *β_d_* are scaling coefficients, *μ_e_* and *μ_d_* define the algebraic saturation strength, *w_e_* and *w_d_* are weighting factors, and *w_f_* is the derivative filter coefficient. These parameters collectively determine the dynamic behavior of the controller, and their interaction results in a complex search space with multiple local optima.

### 4.2. Description of the Objective Function and Optimization Constraints

The objective function plays a key role in guiding the optimization process toward desirable dynamic performance. In this study, a time-domain performance index based on the ZLG (Zhao–Liu–Gao) [52] criterion is adopted to evaluate the control performance of the AVR system.

Unlike classical error-based performance indices such as IAE, ISE, and ITAE, the adopted objective function explicitly incorporates key transient performance measures, including rise time, settling time, overshoot, and steady-state error. Therefore, it provides a more direct and balanced evaluation of the control system’s performance. In this sense, the proposed formulation can be interpreted as a weighted representation of a multi-objective optimization problem, where different performance criteria are simultaneously considered within a single objective function.

The objective function is defined as

(13) $$J\;=\left(1-e^{-\beta }\right)\left(M_{p}+E_{ss}\right)+e^{-\beta }\left(t_{s}-t_{r}\right)$$

where *M_p_*, *E_ss_*, *t_r_* and *t_s_* denote the maximum overshoot, steady-state error, rise time, and settling time, respectively, and *β* is a weighting parameter that controls the trade-off between response speed and damping characteristics. In this study, the weighting parameter *β* is selected as a fixed design parameter and is set to *β* = 1. This value provides a balanced trade-off between response speed and damping characteristics, preventing the objective function from favoring either excessively fast but oscillatory responses or overly slow yet overdamped behavior, as commonly adopted in ZLG-based performance indices [52]. Treating *β* as a fixed parameter also ensures consistency in the evaluation of different controller configurations and avoids increasing the dimensionality of the optimization problem.

The optimization is performed within predefined lower and upper bounds for each decision variable. These bounds ensure the feasibility of the controller parameters and prevent the search process from exploring unstable or non-physical regions of the parameter space.

### 4.3. Animated Oat Optimization Algorithm

The selection of an appropriate optimization algorithm is crucial for AVR controller tuning due to the nonlinear, multimodal, and highly coupled nature of the parameter space. In the proposed ASB-PID structure, the interaction between scaling parameters, saturation parameters, and weighting coefficients creates a complex and irregular search landscape, where small parameter variations may lead to significantly different dynamic responses.

In this context, an effective optimization algorithm must not only explore the global search space but also maintain sufficient diversity to avoid premature convergence and enable fine local adjustments. The Animated Oat Optimization Algorithm (AOOA) is considered suitable for this problem due to its adaptive search behavior, which allows the algorithm to explore the search space broadly in early iterations and progressively refine candidate solutions in later stages.

This capability is particularly important for AVR tuning problems, where both global exploration and precise parameter adjustment are required to achieve a balanced dynamic response. Therefore, AOOA provides an effective framework for tuning the parameters of the proposed nonlinear controller. Based on these considerations, AOOA is adopted in this study for the optimization process.

The AOOA is a population-based metaheuristic inspired by the unique seed dispersal mechanisms of Avena sterilis. Unlike conventional swarm intelligence approaches that primarily rely on social interaction models, AOOA incorporates physically interpretable motion behaviors, including stochastic dispersal, hygroscopic rolling, and energy-driven ejection, to achieve a dynamic balance between global exploration and local exploitation [53].

In AOOA, each candidate solution is represented as a position vector *X_i_* ∈ *R^D^*, where *N* denotes the population size and *D* is the problem dimension. The initial population is randomly generated within the predefined search space as

(14) $$\begin{array}{cc} x_{i,j}\;=\;r_{i,j}\left(UB_{j}-LB_{j}\right)+LB_{j} & i\;=\;1,2,\ldots ,N,j\;=\;1,2,\ldots ,Dim \end{array}$$

where *r_i_*_,_*_j_* ∈ (0,1) and *UB_j_* and *LB_j_* are the upper and lower bounds of the *j*th dimension of the given problem.

To mimic the physical characteristics of seed motion, adaptive parameters are introduced as functions of iteration number. These include the effective mass *m*, characteristic length *L*, eccentricity factor *e*, and a time-varying control coefficient *c*, expressed as

(15) $${\begin{array}{cccc} m\;=\;0.5\times r/\dim, & L\;=\;N\times r/\dim, & e\;=\;0.5\times r/\dim & c\;=\;1-\left(\frac{t}{T}\right) \end{array}}^{3}$$

where *r* is a random number between 0 and 1, dim is the dimensionality of the target problem, *N* is the population size, *L* is the length of the main awn of the Animated Oat seed, *e* is the eccentric rotation coefficient during seed rolling, and *t* and *T* represent the current and maximum iteration numbers, respectively. This formulation allows the algorithm to gradually transition from exploration-dominant behavior to exploitation-oriented refinement.

The global exploration phase models the stochastic dispersal of seeds under environmental influences such as wind and water. The position update is governed by

(16) $$\begin{array}{c} W\;=\;\frac{c}{\pi }\left(2\times r_{\dim}-1\right)⊗UB\\ \left\{\begin{array}{c} X_{t+1}\left(i\right)\;=\;\frac{1}{N}\times \sum_{i=1}^{N}X_{i}^{t}+W,if\;\mathrm{mod}\left(i,N/10\right)=0,\\ X_{t+1}\left(i\right)\;=\;X_{best}+W,if\;\mathrm{mod}\left(i,N/10\right)=1\\ X_{t+1}\left(i\right)\;=\;X_{t}\left(i\right)+W,else. \end{array}\right. \end{array}$$

where *UB* is an upper bound on the target problem, *X_t+1_*(*i*) is the position of the *i*th individual in the *t + 1* generation, and *X_best_* is the position of the best individual in the population. This mechanism enhances diversity and enables broad coverage of the search space.

For exploitation, AOOA introduces two complementary motion models. The first is hygroscopic rolling, which simulates moisture-induced deformation leading to controlled local search. This behavior is modeled as

(17) $$X_{t}^{i}\;=X_{best}+R+c\times Levy\left(\dim\right)⊗X_{best}$$

where

(18) $$R\;=\;\left(m\cdot e+L^{2}\right)\times \frac{r_{\dim}\left(-A,A\right)}{\dim}$$

and *A* is a dynamic boundary parameter. The inclusion of Lévy flight enhances local exploration while maintaining stochasticity.

The second mechanism represents energy-driven ejection, which enables escaping from local optima through a projectile-like motion:

(19) $$X_{t}^{i}\;=X_{best}+J+c\times Levy\left(\dim\right)⊗X_{best}$$

where

(20) $$J\;=\;\frac{2\times k\times x^{2}\times \sin\left(2\theta \right)}{mg}\times \frac{r_{\dim}\left(-B,B\right)}{\dim}\times \left(1-\alpha \right)$$

This mechanism provides strong diversification capability and prevents premature convergence.

At each iteration, candidate solutions are evaluated using the defined objective function, and the global best solution *X_best_* is updated accordingly. The iterative process continues until the termination criterion is satisfied.

The complete procedure of AOOA is summarized in Table 2.

### 4.4. Application of the Optimization Algorithm

**Figure 4:**
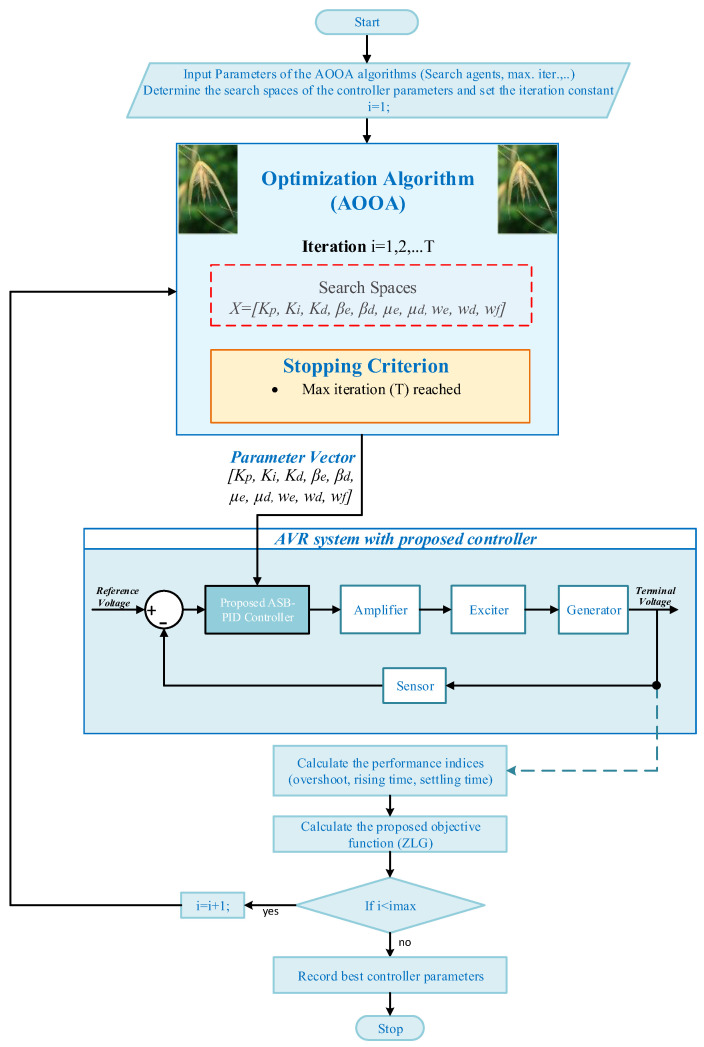
Optimization framework of the proposed ASB-PID controller using AOOA.

The parameter tuning of the proposed ASB-PID controller is carried out using the AOOA within an outer-loop optimization framework, where the algorithm iteratively updates the controller parameters based on the closed-loop dynamic response of the AVR system. The overall optimization structure is illustrated in Figure 4.

In this framework, each individual in the AOOA population represents a candidate solution defined by the parameter vector (*K_p_*, *K_i_*, *K_d_*, *β_e_*, *β_d_*, *μ_e_*, *μ_d_*, *w_e_*, *w_d_*, *w_f_*). These parameters are directly assigned to the ASB-PID controller embedded in the AVR model, and the closed-loop response is obtained under a unit step reference input. The search spaces of all decision variables are predefined to ensure both stability and sufficient exploration capability and are summarized in Table 3. The lower and upper bounds of the decision variables are selected to provide a sufficiently wide but physically feasible search region for the proposed ASB-PID controller. These bounds are determined by considering closed-loop stability requirements, practical controller gain ranges, and the need to avoid excessively aggressive or numerically impractical parameter values. Therefore, the search space represents a physically meaningful domain based on engineering considerations rather than arbitrary numerical limits.

In particular, the derivative filter coefficient $\omega_{f}$ is bounded between 500 and 3000 to provide adequate flexibility for shaping the derivative action in the fast AVR transient response. Lower values of *ω_f_* result in stronger filtering and smoother derivative action, while higher values allow faster derivative response with less attenuation. Therefore, the selected range enables the optimizer to balance noise attenuation and transient responsiveness without allowing an almost unfiltered derivative action. This makes the selected bounds suitable for the considered AVR benchmark system.

From the simulated output voltage, the performance indices *M_p_*, *E_ss_*, *t_r_* and *t_s_* are extracted and substituted into the objective function defined in Section 4.2 to compute the fitness value of each candidate solution. Based on these fitness evaluations, AOOA updates the population through its adaptive search mechanisms and guides the solutions toward improved regions of the search space. This evaluation–update cycle continues until the termination criterion is satisfied, and the best-performing parameter vector is selected as the optimal controller configuration.

All simulations are conducted in the MATLAB/Simulink (R2023b) environment, where the AVR model and the proposed controller are implemented and the optimization process is executed on a high-performance computing platform to ensure numerical stability and consistent convergence behavior.

## 5. Simulation Results

### 5.1. Optimization Results

In this section, the optimization performance of the proposed ASB-PID controller is evaluated using the AOOA and compared with several widely used population-based metaheuristic algorithms, namely PSO, GWO, WOA, and SCA. These algorithms are selected due to their established effectiveness in nonlinear optimization problems and their frequent use in AVR controller tuning studies.

To ensure a fair comparison, all algorithms are executed under the same population size, problem dimension, lower and upper bounds, and maximum iteration number. The parameter settings adopted for each optimization algorithm are summarized in Table 4. For PSO, fixed inertia and acceleration coefficients are used throughout the search process. In both GWO and WOA, the control parameter *a* decreases linearly from 2 to 0 in order to gradually shift the search behavior from exploration to exploitation. Similarly, in SCA, the parameter *r*_1_ is reduced linearly from 2 to 0 to control the step amplitude during the iterative update process. For AOOA, the adaptive parameters are updated dynamically according to the mathematical expressions given in Section 4.3. In addition, each algorithm is executed over 30 independent runs under identical conditions, and the same objective function and performance metrics are used for all methods. Therefore, the convergence curves and statistical analyses are obtained under a unified and fair evaluation framework.

In all simulations, the population size and maximum iteration number are taken as *N* = 50 and *T*_max_ = 200, respectively. The optimized controller parameters obtained by each algorithm are presented in Table 5. It can be observed that different optimizers converge to different parameter combinations, which directly affects the transient response and robustness characteristics of the AVR system. Therefore, beyond the final objective function value, the dynamic behavior produced by the obtained parameter sets should also be examined in detail.

The saturation parameters *μ_e_* and *μ_d_* are dimensionless nonlinear shaping coefficients and should not be interpreted as physical AVR parameters. Their numerical values determine the effective saturation strength of the algebraic nonlinear mapping. In particular, a small value of *μ_d_* indicates that the derivative-related nonlinear channel preserves a wider quasi-linear operating region. This prevents premature saturation of the derivative component and allows useful damping information to be retained during fast transient variations. Therefore, the optimized value of *μ_d_* remains physically meaningful within the proposed control formulation, even when its numerical value is small.

#### Statistical Analysis

**Figure 5:**
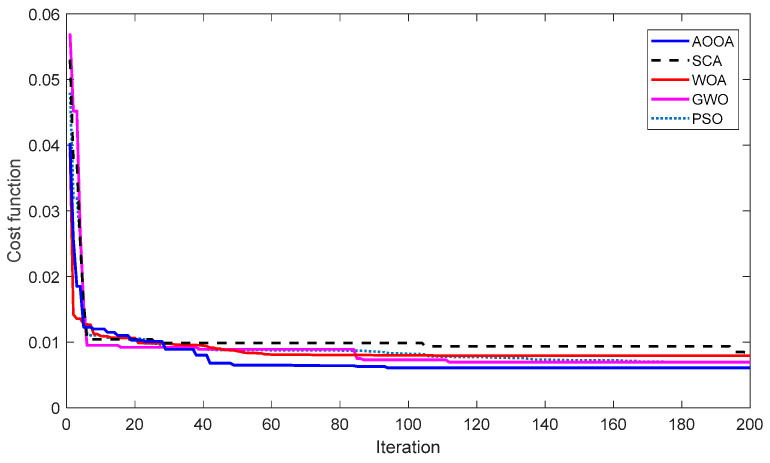
Convergence curves of AOOA compared with benchmark algorithms.

**Figure 6:**
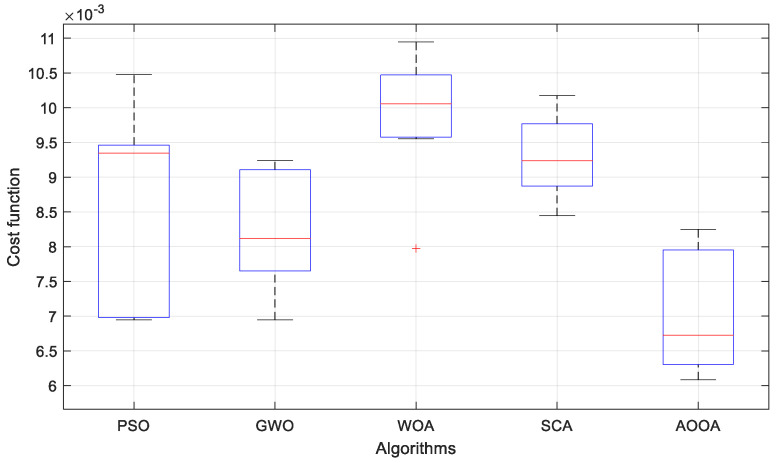
Boxplot of AOOA compared with benchmark algorithms.

To evaluate the consistency and robustness of the employed optimization algorithms, each method is executed over 30 independent runs under identical conditions. The statistical results are summarized in Table 6, including the best, mean, worst, and standard deviation values of the objective function. In addition, the convergence characteristics of the algorithms are illustrated in Figure 5, and the distribution of the obtained results is further analyzed using the boxplot representation in Figure 6.

As observed from Figure 5, AOOA demonstrates a faster convergence rate and reaches a lower objective function value compared to the other algorithms. The convergence curve of AOOA shows a stable and smooth descent without noticeable stagnation, indicating an effective balance between exploration and exploitation. In contrast, GWO exhibits slower convergence and early stagnation behavior, while PSO and SCA show fluctuating convergence patterns. WOA provides relatively stable convergence but fails to reach the same optimal level as AOOA.

The boxplot representation in Figure 6 provides further insight into the distribution characteristics of the optimization results. It can be clearly observed that AOOA exhibits the lowest median value and the narrowest interquartile range among all algorithms, indicating highly stable and consistent performance. The compact distribution of AOOA results confirms that the algorithm reliably converges to high-quality solutions with minimal variability. In contrast, WOA shows a wider spread and noticeable dispersion, reflecting unstable convergence behavior. Similarly, PSO and SCA exhibit broader interquartile ranges and several variations across runs, indicating sensitivity to initial conditions. GWO demonstrates moderate dispersion but still fails to achieve the same level of consistency as AOOA.

The numerical results presented in Table 6 further support these observations. AOOA achieves the lowest best and mean objective values, along with the smallest standard deviation, confirming its superior optimization performance and robustness. The low standard deviation indicates that AOOA maintains consistent performance across different runs, whereas higher deviations observed in the other algorithms reveal their sensitivity to initial conditions and weaker convergence stability.

It should be emphasized that the optimization problem involves a relatively high-dimensional parameter vector consisting of ten decision variables. Despite this complexity, AOOA demonstrates stable convergence behavior without premature stagnation. This indicates that the algorithm is capable of effectively handling high-dimensional and highly coupled optimization problems.

To further validate the statistical significance of the results, the Friedman test is performed, and the average ranking values are reported in Table 7. The results indicate that AOOA attains the best rank among all algorithms with a statistically significant difference (*p* = 4.1033 × 10^−5^ < 0.05), confirming its superiority in solving the AVR controller optimization problem.

A Wilcoxon signed-rank test was conducted and is given in Table 8 to statistically compare the performance of the proposed AOOA with other optimization algorithms.

The Wilcoxon signed-rank test was performed as a post hoc pairwise comparison between AOOA and each competing algorithm. Since all *p*-values are lower than 0.05 and h = 1 for all comparisons, the performance differences between AOOA and the other algorithms are statistically significant.

Overall, both convergence analysis and statistical evaluation demonstrate that AOOA provides the most reliable and consistent optimization performance.

### 5.2. Transient Response Results

To further validate the effectiveness of the proposed AOOA-optimized ASB-PID controller, its dynamic performance is compared with five recently published high-performance AVR controllers reported in the literature. The selected controllers include SFWOA-optimized PID, SFOA-optimized G-PID, NO-optimized PIDA, FWWOA-optimized PIDD^2^, and SOA-optimized FOPID. These methods are chosen due to their recent publication dates and reported superior transient response characteristics. These controllers represent different categories of advanced AVR control strategies. In particular, G-PID introduces additional gain shaping for improved transient performance, FOPID extends the classical PID by incorporating fractional-order dynamics to enhance flexibility, PIDA and PIDD^2^ include higher-order derivative actions for improved damping characteristics, and PID-based structures optimized by recent metaheuristic algorithms reflect modern optimization-driven control design approaches.

The selection of these benchmark controllers is based on their recent publication dates (2024–2026), reported high-performance characteristics, and their relevance in current AVR control research. Therefore, the comparison provides a representative evaluation against state-of-the-art control methods.

For a fair and unbiased comparison, all controller structures are implemented under identical AVR system parameters and simulation conditions. The optimal parameter sets reported in the original studies are directly adopted without modification. The formulations and corresponding parameter values of the considered controllers are summarized in Table 9. The parameter values of the benchmark controllers are adopted from the corresponding literature to preserve their originally reported configurations and ensure consistency with previously published results. Although re-optimizing these controllers for the same AVR model could potentially improve their performance, such an approach would introduce additional variability depending on the selected optimization algorithm and parameter settings.

In this study, all controllers are evaluated under identical simulation conditions using the same AVR model and performance metrics, ensuring a consistent comparison framework. Therefore, the comparison provides a meaningful reference for assessing the relative performance of the proposed controller.

**Figure 7:**
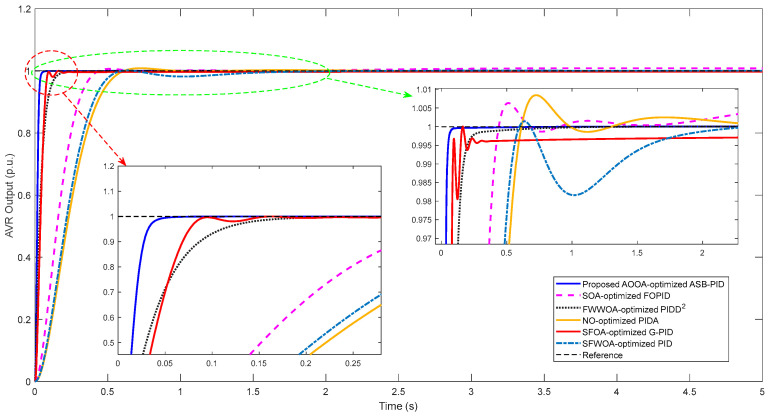
Transient response comparison of the proposed and benchmark controllers.

The transient responses of all controllers under no-load conditions are illustrated in Figure 7, while the corresponding quantitative performance metrics are presented in Table 10. The evaluation is based on rise time (*t_r_*), settling time (*t_s_*), maximum overshoot (*M_p_*), and steady-state error (*E_ss_*).

As clearly observed from Figure 7, the proposed ASB-PID controller exhibits the fastest dynamic response among all compared methods. The response reaches the reference value almost instantaneously with a smooth transition and without any observable oscillatory behavior. In contrast, the other controllers show either slower convergence or noticeable transient oscillations.

The numerical results in Table 10 further confirm these observations. The proposed controller achieves the minimum rise time (*t_r_* = 0.0215 s) and settling time (*t^s^* = 0.0383 s), significantly outperforming all competing methods. For instance, the SFOA-optimized G-PID requires more than twice the settling time, while the NO-optimized PIDA and SOA-optimized FOPID exhibit considerably slower dynamics. This clearly demonstrates the superior speed of the proposed approach.

In terms of overshoot, the proposed ASB-PID achieves a completely overshoot-free response (*Mp* = 0%), whereas several state-of-the-art controllers, such as PIDA and FOPID, exhibit noticeable overshoot. Although some controllers (e.g., G-PID) also achieve zero overshoot, they do so at the expense of slower dynamic performance. Therefore, the proposed controller successfully achieves both zero overshoot and ultra-fast response simultaneously, which is a critical requirement in AVR applications.

Furthermore, the steady-state performance of the proposed controller is also superior. The steady-state error is completely eliminated (*Ess* = 0), while some competing methods, particularly the classical PID-based structure, exhibit small but non-negligible steady-state deviations.

A more detailed inspection of Figure 7 reveals that the proposed ASB-PID effectively suppresses high-frequency oscillations and eliminates residual ripples during the steady-state operation. In contrast, PIDD^2^ and FOPID-based controllers exhibit oscillatory behavior due to the presence of higher-order derivative and fractional dynamics. Similarly, the classical PID structure shows slower convergence and less smooth tracking performance.

The superior performance of the proposed controller can be attributed to its algebraic saturation-based nonlinear structure. This structure enables adaptive sensitivity by providing strong control action during large transient deviations while ensuring smooth regulation near the equilibrium point. As a result, the controller achieves an excellent balance between response speed and stability without introducing additional complexity.

Overall, both graphical and numerical results demonstrate that the AOOA-optimized ASB-PID controller provides the best overall performance in terms of speed, stability, and accuracy among the considered methods. These results clearly validate the effectiveness of the proposed control strategy and justify its superiority over recently developed AVR controllers.

In addition to the transient response characteristics, the control effort of the proposed ASB-PID controller was also examined to assess its practical applicability. It was observed that the control signal remains bounded within a limited range and exhibits rapidly decaying oscillatory behavior during the transient period. Following the transient phase, the control signal quickly converges to a small steady-state value. These observations confirm that the ultra-fast response is achieved without generating excessive or impractical control actions, ensuring compatibility with real-world excitation system constraints.

### 5.3. Robustness Analysis Under Parameter Uncertainties

To further evaluate the robustness of the proposed AOOA-optimized ASB-PID controller, additional simulations are conducted under parameter uncertainty conditions. In this analysis, the time constants of the amplifier (τ_a_), exciter (τ_e_), generator (τ_g_) and sensor (τ_s_) are varied within a wide range of −50% to +50% of their nominal values. This evaluation aims to investigate the capability of the proposed controller to maintain stability and acceptable dynamic performance under significant deviations in system dynamics without requiring any retuning.

**Figure 8:**
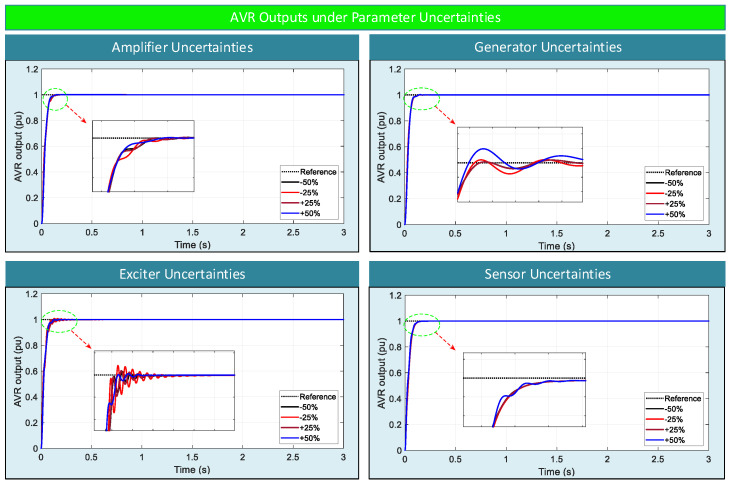
Robustness analysis results under parameter uncertainties.

The corresponding dynamic responses and time-domain performance indices are presented in Figure 8 and Table 11, respectively.

As observed in Figure 8, the AVR output responses remain smooth and stable under all uncertainty scenarios. In the case of amplifier uncertainties, the response is almost unaffected by parameter changes, maintaining a fast transient with zero overshoot. This observation is also supported by Table 11, where the settling time remains approximately around 0.04 s for all cases, and no steady-state error is observed.

For exciter uncertainties, a more noticeable effect on the transient response is observed. As shown in Table 11, the settling time increases up to 0.2130 s under certain conditions, and a small amount of overshoot appears, with a maximum value of 0.8570%. Despite these variations, the system remains stable and the oscillations are rapidly damped, as clearly illustrated in Figure 8.

In the case of generator uncertainties, the controller continues to provide well-damped responses with very small overshoot values. The maximum overshoot is limited to 0.1940%, and the steady-state error remains on the order of 10^−6^, indicating high steady-state accuracy. The settling time remains close to the nominal case, demonstrating that the controller effectively compensates for variations in generator dynamics.

For sensor uncertainties, the transient response is largely preserved with zero overshoot across all tested cases. Although a slight steady-state error appears due to measurement dynamics, its magnitude remains very small (on the order of 10^−4^), which is acceptable for practical AVR applications.

Overall, the results indicate that the proposed ASB-PID controller maintains stable and well-damped performance across all parameter uncertainty scenarios. The variations in transient performance are limited and remain within acceptable bounds, even under significant deviations from nominal conditions.

This behavior demonstrates that the nonlinear algebraic saturation mechanism effectively adapts the control action according to system dynamics, ensuring consistent performance. Furthermore, the controller does not exhibit any instability or performance degradation under extreme parameter variations, confirming that the optimized parameters are not over-tuned to a specific operating point.

These findings clearly verify that the proposed AOOA-optimized ASB-PID controller provides strong robustness against parameter uncertainties and is well-suited for practical AVR applications.

### 5.4. Robustness Analysis Under Load Disturbance

**Figure 9:**
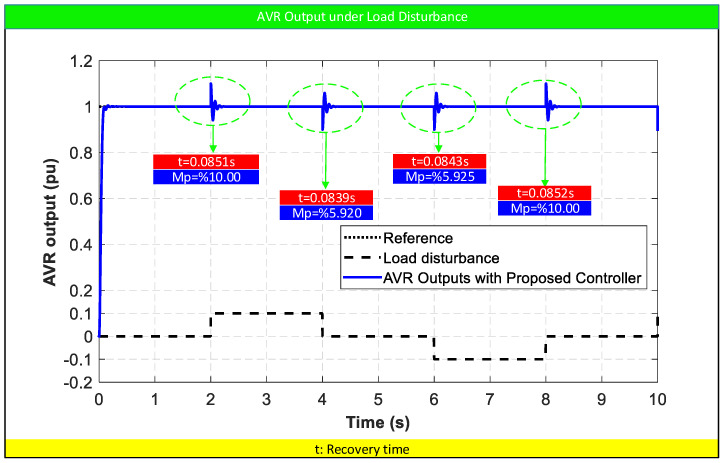
Robustness analysis results under load disturbances.

In addition to parameter uncertainties, the disturbance rejection capability of the proposed controller is evaluated under load variation conditions. In practical AVR systems, sudden load changes can introduce voltage deviations, and the controller is expected to rapidly restore the terminal voltage to its reference value while maintaining stable and well-damped behavior. To assess this capability, a series of step load disturbances are applied to the AVR system at different time instants, as illustrated in Figure 9. The disturbance profile includes both positive and negative variations to simulate realistic operating conditions. The controller performance is evaluated based on recovery time and transient deviation characteristics.

As observed in Figure 9, the proposed ASB-PID controller effectively suppresses the impact of load disturbances and restores the AVR output to the reference value within a very short time interval. The recovery times are consistently around 0.084 s, demonstrating the fast dynamic response of the controller under disturbance conditions.

The transient deviations caused by the disturbances remain limited, with the maximum deviation levels ranging approximately between 5.9% and 10%. Despite these perturbations, the controller ensures a rapid return to steady-state without introducing oscillatory behavior or instability.

It is also noteworthy that the response characteristics remain consistent across successive disturbances, indicating that the controller does not suffer from performance degradation under repeated perturbations. This behavior confirms the strong disturbance rejection capability and stability of the proposed control structure.

Furthermore, the absence of prolonged oscillations or drift after disturbance events highlights the effectiveness of the algebraic saturation mechanism in regulating control effort during transient conditions. The controller dynamically adjusts its sensitivity, enabling fast correction while avoiding excessive control action.

Overall, the results demonstrate that the proposed AOOA-optimized ASB-PID controller provides excellent disturbance rejection performance, ensuring rapid recovery, limited deviation, and stable operation under varying load conditions. This makes the proposed approach highly suitable for real-world AVR applications where load disturbances are unavoidable.

### 5.5. Performance Benchmarking with Cutting-Edge AVR Control Designs

To further demonstrate the effectiveness and generalization capability of the proposed controller, a comprehensive benchmarking study is conducted against recently published cutting-edge AVR control designs. Unlike the previous section, where identical system structures were considered, this analysis incorporates different controller architectures and optimization strategies reported in the literature, thereby providing a more realistic and challenging comparison scenario.

The selected benchmark methods, denoted as M1–M11, cover a broad spectrum of advanced control strategies, including G-PID, PIDN, FOPID, fuzzy-based hybrid controllers, and higher-order derivative-based structures. The associated optimization algorithms include SFOA, Adaptive and Dynamic Inertia Weight and Acceleration Coefficient Optimization (ADIWACO), Artificial Hummingbird Algorithm (AHA), PSO, Teaching-Learning-Based Optimization (TLBO), Black-Winged Kite Optimization Algorithm (BWOA), NSCA-SED, Snake Optimization (SO), Modified Opposition Learning based Weighted Mean of Vectors (mOBL-INFO), Harmony Search And Dwarf Mongoose Optimization Algorithms (HS-DMOA), and Quadratic Wavelet-Enhanced Gradient-Based Optimization (QWGBO), representing some of the most recent developments in AVR controller optimization. The detailed information regarding these methods is summarized in Table 12.

**Figure 10:**
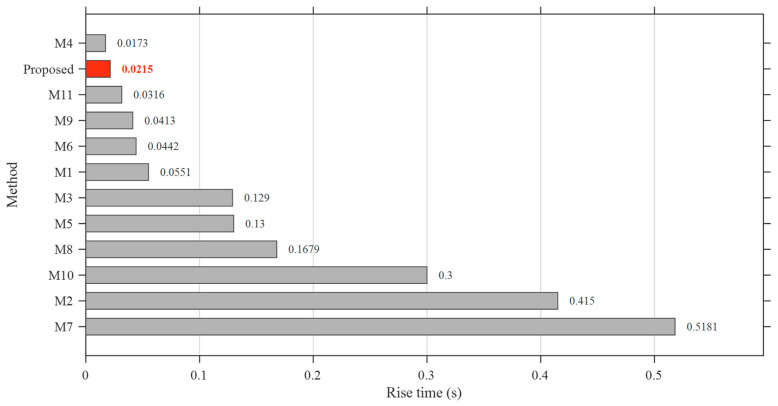
Comparison of rise time performance of the proposed ASB-PID controller and benchmark AVR control methods (M1–M11).

**Figure 11:**
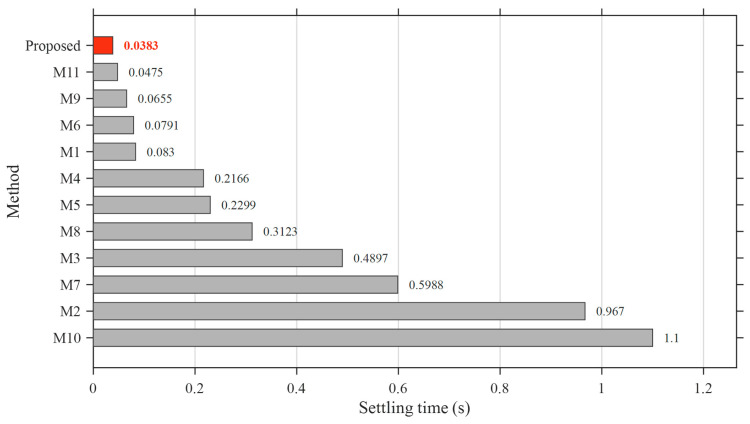
Comparison of settling time performance of the proposed ASB-PID controller and benchmark AVR control methods (M1–M11).

**Figure 12:**
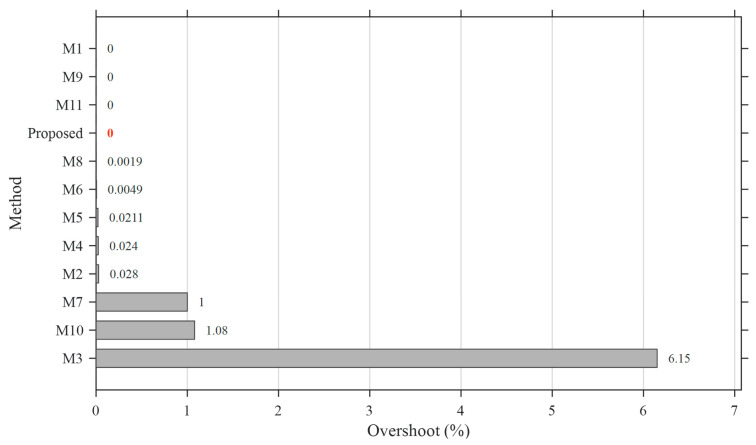
Comparison of overshoot characteristics of the proposed ASB-PID controller and benchmark AVR control methods (M1–M11).

**Figure 13:**
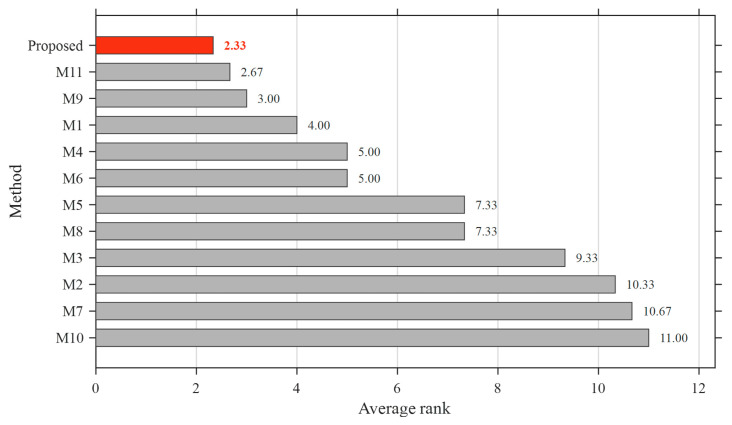
Overall performance ranking of the proposed ASB-PID controller and benchmark AVR control methods based on transient response metrics.

The comparative performance is evaluated in terms of rise time, settling time, overshoot, and overall ranking, as illustrated in Figure 10, Figure 11, Figure 12 and Figure 13.

The transient performance metrics of the benchmark methods (M1–M11) are directly adopted from the corresponding original publications, where the results are explicitly reported in tabular form. This approach ensures high accuracy and consistency with the reported literature results while avoiding potential errors associated with data digitization.

The results reveal that the proposed AOOA-optimized ASB-PID controller consistently delivers superior performance across all considered metrics. In terms of rise time, the proposed method achieves a very fast response (*t_r_* = 0.0215 s), placing it among the best-performing controllers. Although one method (M4) exhibits a slightly faster rise time, this marginal improvement is achieved at the expense of degraded performance in other metrics, indicating an inherent trade-off.

A more significant advantage of the proposed controller becomes evident in the settling time analysis. The ASB-PID achieves the lowest settling time (*t_s_* = 0.0383 s) among all benchmarked methods, demonstrating its capability to reach steady-state conditions rapidly and efficiently. Even the closest competitors, such as M11 and M9, require noticeably longer settling durations, while several methods exhibit considerably slower dynamics, with settling times approaching or exceeding 1 s.

The overshoot analysis further highlights the robustness of the proposed approach. The ASB-PID achieves a completely overshoot-free response (*Mp* = 0%), placing it among the best-performing methods in terms of stability. While a few methods also exhibit zero overshoot, they generally suffer from slower transient behavior. In contrast, several advanced controllers, including M3, M7, and M10, produce significant overshoot, which is undesirable in AVR applications due to potential voltage stress and reduced system reliability.

In addition to individual performance metrics, an overall ranking based on transient response characteristics is presented, which clearly indicates that the proposed controller achieves the best (lowest) average rank among all compared methods. Many of the recently developed controllers, despite their structural complexity and increased number of parameters, rank significantly lower due to their inability to simultaneously achieve fast response, low settling time, and minimal overshoot.

The superior performance of the proposed controller can be attributed to its algebraic saturation-based nonlinear structure, which enables adaptive control action depending on the magnitude of the error signal. This feature allows the controller to apply aggressive control during large transient deviations while maintaining smooth and precise regulation near the steady-state region. When combined with the strong global search capability of AOOA, this results in an effective balance between speed, stability, and accuracy.

Moreover, it is important to emphasize that the proposed ASB-PID controller achieves these performance improvements without introducing excessive complexity. Compared to hybrid, fuzzy, and fractional-order controllers, the proposed method maintains a relatively moderate number of parameters, which enhances its practical applicability and implementation feasibility.

Overall, the results of this comprehensive benchmarking study clearly demonstrate that the AOOA-optimized ASB-PID controller outperforms recently developed high-performance AVR control strategies and establishes a new benchmark in terms of transient response speed, stability, and overall control quality.

## 6. Real-Time Implementation Scheme of the Proposed Controller

**Figure 14:**
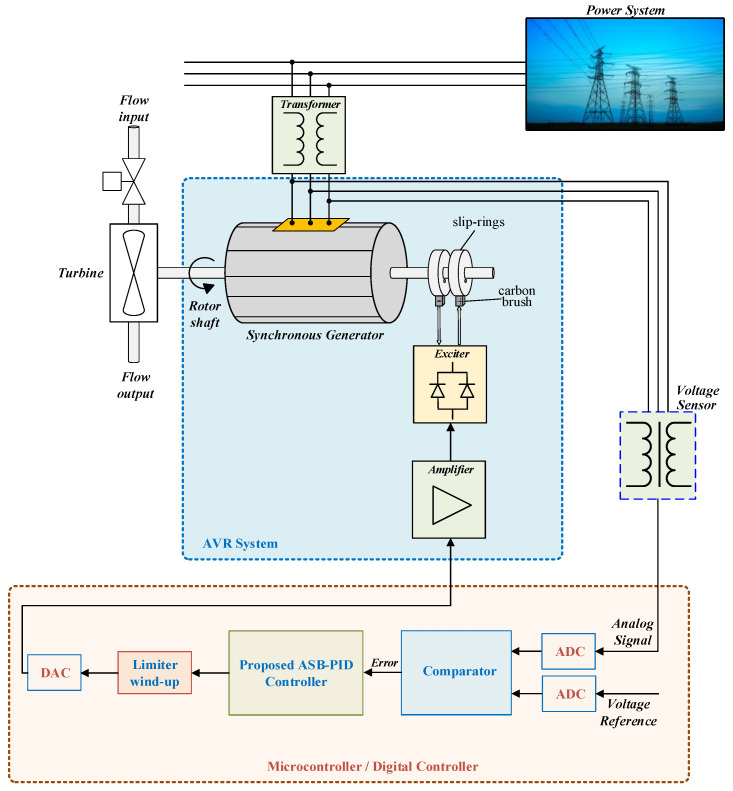
Real-time implementation scheme of proposed ASB-PID controller for AVR system.

The real-time implementation scheme of the proposed ASB-PID controller for the AVR system is illustrated in Figure 14. The control structure is based on a microcontroller-oriented architecture, enabling direct deployment in practical excitation control systems.

In this configuration, the terminal voltage of the synchronous generator is measured through a voltage sensing unit and conditioned before being sampled by the analog-to-digital converter (ADC). The reference voltage is also digitized and compared with the measured signal to generate the error signal e(t). This error signal is processed by the proposed ASB-PID controller implemented in discrete-time form.

The nonlinear algebraic saturation functions embedded in the controller are evaluated at each sampling instant using the sampled signals. Due to their analytical structure, these functions require only basic arithmetic operations and a square-root computation, which makes them suitable for real-time execution on standard microcontroller platforms without introducing significant computational overhead.

The control signal generated by the controller is subjected to a limiting mechanism with anti-windup protection to prevent integrator accumulation under actuator saturation conditions. This is particularly important in practical AVR systems where excitation voltage constraints are present. The limited control signal is then converted into an analog signal using a digital-to-analog converter (DAC) and applied to the excitation system through the amplifier.

From an implementation perspective, the derivative component is realized using a first-order filter, which plays a critical role in attenuating high-frequency measurement noise. The selection of the derivative filter coefficient should be carefully performed by considering the sampling frequency and sensor noise characteristics. In practice, excessively large values may amplify measurement noise, whereas overly small values may degrade transient performance.

It is important to emphasize that the optimization process used to determine the controller parameters is performed offline. Therefore, the real-time operation involves only the execution of the controller equations, significantly reducing computational complexity. As a result, the proposed ASB-PID controller maintains a computational burden comparable to that of a conventional PID controller, with only a marginal increase due to the nonlinear saturation operations.

Finally, the presented implementation structure ensures compatibility with practical AVR systems and demonstrates that the proposed controller can be effectively deployed in real-time environments while preserving robustness, fast dynamic response, and stable operation under practical constraints.

## 7. Discussion

The results presented in this study provide a comprehensive evaluation of the proposed AOOA-optimized ASB-PID controller from multiple perspectives, including optimization performance, transient response characteristics, and benchmarking against recently developed AVR control strategies. The findings consistently demonstrate that the proposed approach achieves superior performance in terms of response speed, stability, and robustness.

From an optimization standpoint, the statistical analysis reveals that AOOA exhibits strong global search capability and reliable convergence behavior. The algorithm achieves the lowest objective function values while maintaining a highly consistent performance across multiple independent runs. The convergence characteristics further indicate that AOOA effectively balances exploration and exploitation, avoiding premature convergence and ensuring a stable search process. In addition, the compact distribution observed in the boxplot analysis confirms that the algorithm reliably converges to high-quality solutions with minimal variability, highlighting its robustness against random initialization.

In terms of control performance, the proposed ASB-PID controller introduces a nonlinear algebraic saturation mechanism that significantly enhances control sensitivity across different operating regions. This mechanism enables aggressive control action during large transient deviations while ensuring smooth and precise regulation near the steady state. As a result, the proposed controller achieves ultra-fast rise and settling times without overshoot and eliminates oscillatory behavior, which are critical requirements for AVR systems.

The comparative analyses clearly demonstrate that the proposed approach outperforms both classical and recently developed control strategies. Unlike many existing methods that improve one performance metric at the expense of others, the ASB-PID controller achieves a well-balanced performance across all key criteria. In particular, it simultaneously ensures fast dynamic response, zero overshoot, and high steady-state accuracy. This balanced performance is rarely achieved by conventional PID, fractional-order, or hybrid control structures, which often suffer from trade-offs between speed and stability.

Another important advantage of the proposed controller is its structural efficiency. Compared to advanced controllers such as FOPID, PIDD^2^, and fuzzy-based hybrid designs, the ASB-PID maintains a relatively moderate number of tunable parameters while delivering superior performance. This characteristic reduces the design complexity and computational burden, making the proposed controller more suitable for practical implementations in real-world AVR systems.

The proposed control framework also offers significant flexibility for further extensions. Owing to its modular structure, the ASB-PID controller can be readily adapted to more complex and highly nonlinear power system scenarios, including renewable energy-integrated grids and disturbance-rich environments. In addition, the computational efficiency and structural simplicity of the proposed approach make it suitable for real-time implementation and hardware-in-the-loop (HIL) validation. These aspects indicate that the proposed method is not only effective for the considered AVR system but also has strong potential for broader practical applications.

Overall, the results position the proposed AOOA-optimized ASB-PID controller as a highly effective and competitive solution for AVR control design, offering a compelling combination of fast dynamic response, robustness, and implementation efficiency.

## 8. Conclusions and Future Works

In this study, a novel ASB-PID controller optimized by the AOOA is proposed for AVR systems. The proposed controller integrates a nonlinear algebraic saturation mechanism into the classical PID structure, enabling adaptive control sensitivity across different operating regions. This formulation enhances transient performance while preserving structural simplicity and practical applicability.

The effectiveness of the proposed approach is validated through a comprehensive evaluation framework. First, the optimization capability of AOOA is assessed and compared with widely used metaheuristic algorithms, including PSO, GWO, WOA, and SCA. The statistical results demonstrate that AOOA achieves superior performance in terms of solution quality, convergence speed, and robustness. Subsequently, the dynamic performance of the proposed controller is evaluated and compared with both classical and recently developed AVR control strategies.

Simulation results reveal that the proposed AOOA-optimized ASB-PID controller achieves a rise time of 0.0215 s and a settling time of 0.0383 s, with zero overshoot and negligible steady-state error. Compared to the benchmark controllers, the proposed method provides substantial performance improvements. In particular, the settling time is reduced by approximately 60–80% compared to several state-of-the-art methods, while the rise time is improved by approximately 40–65%, depending on the reference controller. In addition, the proposed controller eliminates overshoot entirely, whereas existing methods exhibit overshoot levels ranging from approximately 0.5% to 10%. These results clearly demonstrate the effectiveness of the proposed approach in achieving fast and well-damped transient responses.

The superior performance of the proposed method is mainly attributed to the combination of the algebraic saturation-based nonlinear control structure and the strong global search capability of AOOA. In contrast to conventional nonlinear PID designs based on hyperbolic tangent, sigmoid, or Gudermannian functions, the proposed algebraic saturation mechanism provides a more explicitly controllable and stronger saturation behavior, enabling faster attenuation of large transient deviations while preserving high sensitivity near the equilibrium region. This characteristic allows the controller to achieve a more effective balance between fast dynamic response and stable steady-state behavior without increasing system complexity.

Overall, the proposed AOOA-optimized ASB-PID controller provides a robust, efficient, and high-performance solution for AVR systems. In addition, the proposed control framework offers a scalable structure that can be extended to other nonlinear control applications, making it a promising candidate for next-generation control system design.

Future work will focus on the experimental validation of the proposed ASB-PID controller on a real-time AVR testbed to assess its performance under practical operating conditions. In addition, the extension of the proposed control framework to multi-machine power systems will be investigated to evaluate its effectiveness in large-scale interconnected environments. Furthermore, the integration of the proposed controller with renewable energy-based power systems, such as photovoltaic and wind energy systems, will be explored to address emerging challenges in modern power grids.

## Figures and Tables

**Table 1:** AVR system parameters and their operating ranges.

Component	Range of the Gain	Range of the Time Constant (s)	Gain	Time Constant (s)
Amplifier	10 ≤ K_a_ ≤ 40	0.02 ≤ τ_a_ ≤ 0.1	K_a_ = 10	τ_a_ = 0.1
Exciter	1.0 ≤ K_e_ ≤ 10	0.1 ≤ τ_e_ ≤ 0.4	K_e_ = 1.0	τ_e_ = 0.4
Generator	0.7 ≤ K_g_ ≤ 1.0	1.0 ≤ τ_g_ ≤ 2.0	K_g_ = 1.0	τ_g_ = 1.0
Sensor	0.9 ≤ K_s_ ≤ 1.1	0.001 ≤ τ_s_ ≤ 0.06	K_s_ = 1.0	τ_s_ = 0.01

**Table 2:** Pseudo code of the AOOA.

Step	Description
1	Generate the initial population *X_i_* within [*LB*,*UB*] and evaluate fitness.
2	while *t* ≤ *T_max_*
3	For *i = 1:N*
4	Calculate the parameters according to Equation (15);
5	If *r*_1_ > 0.5 (Exploration phase):
6	Update the position according to Equation (16);
7	Else
8	If *r*_2_ > 0.5 (Rolling mechanism):
9	Update the position $X_{i}^{t+1}=X_{best}+J+c\cdot Levy\left(D\right)$
10	Else
11	Update the position according to Equations (19) and (20);
12	end if
13	end if
14	Calculate the fitness value for each individual;
15	end for
16	Record the best position and fitness values for population;
17	end while

**Table 3:** Search space of the controller parameters.

	*K_p_*	*K_i_*	*K_d_*	*β_e_*	*β_d_*	*µ_e_*	*µ_d_*	*ω_e_*	*ω_d_*	*ω_f_*
Lower	0.1	0.1	0.1	0.5	0.01	1	0.001	1	0.5	500
Upper	30	25	20	2	0.05	5	0.1	5	5	3000

**Table 4:** Control parameters of the optimization algorithms.

Algorithm	Control Parameters	Value/Description
AOOA	Adaptive motion parameters	updated dynamically
PSO	Inertia weight *ω*	0.8
	Cognitive coefficient *c*_1_	1.49445
	Social coefficient *c*_2_	1.49445
GWO	Control parameter *a*	Linearly decreased from 2 to 0
WOA	Control parameter *a*	Linearly decreased from 2 to 0
	Spiral constant *b*	1
SCA	Control parameter *r*_1_	Linearly decreased from 2 to 0

**Table 5:** Optimized parameters of the ASB-PID controller obtained by different optimization algorithms.

Algorithm	*K_p_*	*K_i_*	*K_d_*	*β_e_*	*β_d_*	*µ_e_*	*µ_d_*	*ω_e_*	*ω_d_*	*ω_f_* (Rad/s)
AOOA	29.1221	23.2434	13.0084	1.8911	0.0260	3.1553	0.0031	3.7704	2.9690	2496.52
SCA	27.1172	24.9814	13.7486	1.5588	0.0206	3.9944	0.0087	3.1646	3	1501.31
PSO	15.0812	24.7768	13.7184	2	0.03	2.6121	0.001	3.0578	2.3205	2266.78
WOA	22.8803	21.1763	13.1769	1.8360	0.0289	3.7323	0.0075	4.3650	2.7905	2294.90
GWO	22.6229	22.5243	12.5052	1.6298	0.0268	3.9462	0.0033	4.0851	2.8393	2307.29

**Table 6:** Statistical results of the optimization algorithms.

Algorithm	Best	Mean	Worst	Std
AOOA	**6.0858 × 10^−3^**	**7.0605 × 10^−3^**	**8.2513 × 10^−3^**	0.8288 × 10^−3^
SCA	8.4450 × 10^−3^	9.2812 × 10^−3^	10.1736 × 10^−3^	**0.5319 × 10^−3^**
PSO	6.9454 × 10^−3^	8.7549 × 10^−3^	10.4756 × 10^−3^	1.2880 × 10^−3^
WOA	7.9741 × 10^−3^	9.9331 × 10^−3^	10.9488 × 10^−3^	0.8103 × 10^−3^
GWO	6.9491 × 10−3	8.1456 × 10^−3^	9.2391 × 10^−3^	0.8262 × 10^−3^

**Table 7:** Friedman average ranking values.

Algorithm	Mean Rank
AOOA	**1.3**
SCA	3.7
PSO	3.3
WOA	4.5
GWO	2.2
*p* = 4.1033 × 10^−5^ Significance level = 0.05	

**Table 8:** Pairwise Wilcoxon Signed-Rank Test Results.

Pairwise Comparison	*p*-Value	h	Interpretation
AOOA vs. PSO	0.005859375	1	Significant
AOOA vs. GWO	0.02734375	1	Significant
AOOA vs. WOA	0.001953125	1	Significant
AOOA vs. SCA	0.001953125	1	Significant

**Table 9:** Overview of recently reported AVR controllers, including their structures, optimization methods, and corresponding parameter configurations.

Ref.	Year	Controller Type	Optimization Algorithm	Number of Parameters	Parameter Values
[48]	2026	PID	SFWOA	3	K_P_ = 0.5914, K_I_ = 0.4078, K_D_ = 0.1954
[36]	2026	G-PID	SFOA	8	K_P_ = 6.7184, K_I_ = 0.5812, K_D_ = 4.0806, η = 983.8587, τ_1_ = 4.5068, G_1_ = 0.9032, τ_2_ = 0.0556, G_2_ = 1.6840
[13]	2025	PIDA	NO	6	K_P_ = 777.98, K_I_ = 365.18, K_D_ = 506.09, K_A_ = 111.586, α = 623.3, β = 903.97
[14]	2024	PIDD^2^	FWWOA	4	K_P_ = 2.9743, K_I_ = 1.9827, K_D_ = 1.0705, K_D2_ = 0.0790
[17]	2025	FOPID	SOA	5	K_P_ = 0.9697, K_I_ = 0.4918, K_D_ = 0.2210, λ = 1.1522, µ = 1.1524

**Table 10:** Transient response performance comparison of the proposed and benchmark AVR controllers.

Controller	Rising Time (s)	Settling Time (s)	Overshoot (%)	Steady-State Error
Proposed AOOA-optimized ASB-PID	0.0215	0.0383	**0**	**0**
SFOA-optimized G-PID	0.0551	0.0830	0	0
FWWOA-optimized PIDD^2^	0.0848	0.1484	0.0033	0
NO-optimized PIDA	0.185	0.6120	1.0622	0
SFWOA-optimized PID	0.3300	0.5140	0.0230	1 × 10^−3^
SOA-optimized FOPID	0.2483	0.3889	0.5446	0.272 × 10^−3^

**Table 11:** Time-domain specifications of the AVR output with the proposed ASB-PID controller under parameter uncertainties.

AVR Component	Time Constant Change	Rising Time (s)	Settling Time (s)	Overshoot (%)	Steady-State Error
Amplifier $\left(\tau_{a}\right)$	−50%	0.0228	0.0394	0	0
	−25%	0.0207	0.0485	0	0
	+25%	0.0211	0.0396	0	0
	+50%	0.0219	0.0391	0	0
Exciter $\left(\tau_{e}\right)$	−50%	0.0308	0.1597	0.3140	0
	−25%	0.0356	0.2130	0.8570	0
	+25%	0.0311	0.1570	0.3930	0
	+50%	0.0288	0.0955	0.2500	0
Generator ${(\tau }_{g})$	−50%	0.0222	0.0408	0.0255	1.70 × 10^−6^
	−25%	0.0225	0.0411	0.0430	1.45 × 10^−6^
	+25%	0.0214	0.0399	0.0250	1.55 × 10^−6^
	+50%	0.0209	0.0396	0.1940	5.00 × 10^−6^
Sensor ($\tau_{s})$	−50%	0.0219	0.0395	0	1.02 × 10^−4^
	−25%	0.0217	0.0393	0	1.15 × 10^−4^
	+25%	0.0218	0.0397	0	1.50 × 10^−4^
	+50%	0.0206	0.0402	0	1.38 × 10^−4^

**Table 12:** Summary of recently published cutting-edge AVR control designs, including controller structures and associated optimization algorithms.

Method	Ref.	Year	Controller Type	Optimization Algorithm
M1	[36]	2026	G-PID	SFOA
M2	[2]	2025	PIDN	ADIWACO
M3	[40]	2025	FOPID	AHA
M4	[27]	2025	Fuzzy PIDF + FOPD	PSO
M5	[28]	2025	PIDF + Fuzzy PIDF	TLBO
M6	[21]	2025	FOPIDD^2^	BWOA
M7	[35]	2025	SPID	NSCA-SED
M8	[8]	2025	ePIDA	SO
M9	[59]	2025	rPIDD^2^	mOBL-INFO
M10	[45]	2024	PID	HS-DMOA
M11	[29]	2024	RPIDD^2^-FOPI	QWGBO

## Data Availability

All data supporting the findings of this study are included within the manuscript.

## References

[1] Amin M.S., Attia M.A., Khamees A.K., Mekhamer S.F., Kotb H., AboRas K.M., Yousef A. (2024). Development of AVR controller performance using exponential distribution and transit search optimization techniques. Front. Energy Res..

[2] Sekyere Y.O.M., Ajiboye P.O., Effah F.B., Opoku B.T. (2025). Optimizing PID control for automatic voltage regulators using ADIWACO PSO. Sci. Afr..

[3] Liu S., Lin Z., Feng R., Huang W., Yan B. (2025). Intelligent control method for automatic voltage regulator: An improved coati optimization algorithm-based strategy. Measurement.

[4] Ji T., Lin P., Zhu M., Jiang W., Zhang X., Wen C., Wang P. (2025). Mode-Constrained Two-Stage Current Injection Optimization for Voltage Unbalance Mitigation in IBR-Rich Distribution Network. IEEE J. Emerg. Sel. Top. Power Electron..

[5] Yang D., Yuan X., Gao H., Ma J., Chen Z. (2025). FFRLS-Based Data-Driven Voltage Security Assessment for Active Distribution Networks. IEEE Trans. Smart Grid.

[6] Saka M. (2024). Novel hVsaGwo algorithm for non-linear dynamic weighted state feedback with 1DOF-PID based controllers in AVR. Eng. Sci. Technol. Int. J..

[7] Izci D., Hashim F.A., Ekinci S., Sabbeh S.F., Bajaj M., Prokop L., Zaitsev I. (2025). A novel cascaded RPIDD2-PI controller tuned by enhanced cooperation search algorithm for automatic voltage regulator systems. IET Control Theory Appl..

[8] Chetty N.D., Gandhi R., Sharma G., Çelik E., Kumar R. (2025). Enhanced automatic voltage regulation using an extended PIDA controller optimised by the snake algorithm. Results Eng..

[9] Padiachy V., Mehta U., Azid S., Prasad S., Kumar R. (2022). Two degree of freedom fractional PI scheme for automatic voltage regulation. Eng. Sci. Technol. Int. J..

[10] Gozde H. (2020). Robust 2DOF state-feedback PI-controller based on meta-heuristic optimization for automatic voltage regulation system. ISA Trans..

[11] Eke I., Saka M., Gozde H., Arya Y., Taplamacioglu M.C. (2021). Heuristic optimization based dynamic weighted state feedback approach for 2DOF PI-controller in automatic voltage regulator. Eng. Sci. Technol. Int. J..

[12] Mosaad A.M., Attia M.A., Abdelaziz A.Y. (2019). Whale optimization algorithm to tune PID and PIDA controllers on AVR system. Ain Shams Eng. J..

[13] Mosaad A.M., Attia M.A., Elbehairy N.M., Alruwaili M., Yousef A., Hamed N.M. (2025). Enhancing AVR System Stability Using Non-Monopolize Optimization for PID and PIDA Controllers. Processes.

[14] Dai F., Gao S. (2024). Optimal Design of a PIDD2 Controller for an AVR System Using Hybrid Whale Optimization Algorithm. IEEE Access.

[15] Dursun E.H. (2024). Optimal FOPID and RPIDD2 Controller Design Using Meta-Heuristic Optimization for AVR System. Proceedings of the 2024 8th International Symposium on Multidisciplinary Studies and Innovative Technologies (ISMSIT).

[16] Sikander A., Thakur P., Bansal R.C., Rajasekar S. (2018). A novel technique to design cuckoo search based FOPID controller for AVR in power systems. Comput. Electr. Eng..

[17] Jegatheesh A., Thiyagarajan V., Selvan N.M., Raj M.D. (2024). Voltage regulation and stability enhancement in AVR system based on SOA-FOPID controller. J. Electr. Eng. Technol..

[18] Ayas M.S., Sahin E. (2021). FOPID controller with fractional filter for an automatic voltage regulator. Comput. Electr. Eng..

[19] Mok R., Ahmad M.A. (2022). Fast and optimal tuning of fractional order PID controller for AVR system based on memorizable-smoothed functional algorithm. Eng. Sci. Technol. Int. J..

[20] Munagala V.K., Jatoth R.K. (2022). Improved fractional PIλDμ controller for AVR system using Chaotic Black Widow algorithm. Comput. Electr. Eng..

[21] Dai F., Ma T., Gao S. (2025). Optimal design of a fractional order PIDD2 controller for an AVR system using hybrid black-winged kite algorithm. Electronics.

[22] Izci D., Rizk-Allah R.M., Snášel V., Ekinci S., Migdady H., Daoud M.S., Altalhi M., Abualigah L. (2024). Refined sinh cosh optimizer tuned controller design for enhanced stability of automatic voltage regulation. Electr. Eng..

[23] Başak H. (2026). Efficient Fusion-Based Optimization of Fractional-Order PID Plus Double-Derivative Controller for Automatic Voltage Regulation. Arab. J. Sci. Eng..

[24] Can Ö., Andiç C., Ekinci S., Izci D. (2023). Enhancing transient response performance of automatic voltage regulator system by using a novel control design strategy. Electr. Eng..

[25] Izci D., Abualigah L., Can Ö., Andiç C., Ekinci S. (2024). Achieving improved stability for automatic voltage regulation with fractional-order PID plus double-derivative controller and mountain gazelle optimizer. Int. J. Dyn. Control.

[26] Mahapatra M.D., Konar K., Mahato S., Kumar V., Dey P. Fuzzy PID Controller Design for Enhanced Voltage Regulation in Automatic Voltage Regulator System. Proceedings of the 2024 IEEE International Conference on Information Technology, Electronics and Intelligent Communication Systems (ICITEICS).

[27] Shouran M., Alenezi M., Muftah M.N., Almarimi A., Abdallah A., Massoud J. (2025). A novel AVR system utilizing fuzzy PIDF enriched by FOPD controller optimized via PSO and Sand Cat Swarm Optimization algorithms. Energies.

[28] Shouran M., Alenazi M. (2025). A novel fuzzy PIDF enhancing PIDF controller tuned in two stages by TLBO and PSO algorithms for reliable AVR performance. IEEE Access.

[29] Ekinci S., Snášel V., Rizk-Allah R.M., Izci D., Salman M., Youssef A.A. (2024). Optimizing AVR system performance via a novel cascaded RPIDD2-FOPI controller and QWGBO approach. PLoS ONE.

[30] AboRas K.M., El-Banna M.H., El-Wakil A.M., Hammad M.R. (2025). Optimized cascaded regulation strategy for robust automatic generation control in renewable-integrated power networks. Sci. Rep..

[31] Furat M., Cücü G.G. (2022). Design, implementation, and optimization of sliding mode controller for automatic voltage regulator system. IEEE Access.

[32] Türksoy Ö., Türksoy A. (2024). A fast and robust sliding mode controller for automatic voltage regulators in electrical power systems. Eng. Sci. Technol. Int. J..

[33] Mahal A.A., Hadi A.R.S. (2024). Robust control for automatic voltage regulator system based on learning sliding mode control. Int. J. Adv. Mechatron. Syst..

[34] Furat M. (2023). Chattering attenuation analysis in variable structure control for automatic voltage regulator with input constraints. Eng. Sci. Technol. Int. J..

[35] Tumari M.Z.M., Suid M.H., Ahmad M.A. (2025). Hybrid nonlinear sine cosine and safe experimentation dynamics algorithm for robust sigmoid PID control of automatic voltage regulators. J. King Saud Univ. Comput. Inf. Sci..

[36] Izci D., Ekinci S., Jabari M., Kocaman B., Güneş B.B., Adas E., Ahmad M.A. (2025). A Novel Gudermannian Function-Driven Controller Architecture Optimized by Starfish Optimizer for Superior Transient Performance of Automatic Voltage Regulation. Biomimetics.

[37] Ekinci S., Izci D., Tümen V., Jabari M., Çelik E., Elrashidi A. (2026). Intelligent Control of Magnetic Ball Suspension Systems via a Novel Hyperbolic Tangent PID Controller Tuned by the Artificial Lemming Algorithm. Biomimetics.

[38] Ekinci S., Izci D., Jabari M., Çelik E., Bajaj M., Vishnuram P., Rubanenko O. (2026). A novel hyperbolic tangent-based PID controller tuned by the artificial lemming algorithm for nonlinear steam condenser pressure control. Sci. Rep..

[39] Xiang K., Song Y., Ioannou P. (2025). Nonlinear adaptive PID control for nonlinear systems. IEEE Trans. Autom. Control.

[40] Bouguenna E., Ladaci S., Lekouaghet B., Merrouche W., Benghanem M. (2025). Fractional order PID controller design for an AVR system using the artificial hummingbird optimizer algorithm. Int. J. Robust Nonlinear Control.

[41] Xue Q., Wu C., Nie J., Zhou S., Liu H., Katsikis V.N. (2026). An improved multi-objective animated oat optimization algorithm for resource-constrained construction project organization design. Sci. Rep..

[42] Xiao Y., Cui H., Khurma R.A., Castillo P.A. (2025). Artificial lemming algorithm: A novel bionic meta-heuristic technique for solving real-world engineering optimization problems. Artif. Intell. Rev..

[43] Bhullar A.K., Kaur R., Sondhi S. (2020). Enhanced crow search algorithm for AVR optimization: AK Bhullar et al. Soft Comput..

[44] Moschos I., Parisses C. (2022). A novel optimal PIλDND2N2 controller using coyote optimization algorithm for an AVR system. Eng. Sci. Technol. Int. J..

[45] Hesham O.M., Attia M.A., Mekhamer S.F. (2024). Enhancement of AVR system performance by using hybrid harmony search and dwarf mongoose optimization algorithms. Sci. Rep..

[46] Ali A.K. (2024). An optimal design for an automatic voltage regulation system using a multivariable PID controller based on hybrid simulated annealing–white shark optimization. Sci. Rep..

[47] Çavdar B., Şahin E., Akyazı Ö., Nuroğlu F.M. (2023). A novel optimal PIλ1Iλ2Dμ1Dμ2 controller using mayfly optimization algorithm for automatic voltage regulator system. Neural Comput. Appl..

[48] Andiç C. (2026). A Novel Superb Fairy-Wren Optimization Algorithm Based PID Controller for an Automatic Voltage Regulator System. Appl. Sci..

[49] Mirjalili S., Mirjalili S.M., Lewis A. (2014). Grey wolf optimizer. Adv. Eng. Softw..

[50] Mirjalili S., Lewis A. (2016). The whale optimization algorithm. Adv. Eng. Softw..

[51] Mirjalili S. (2016). SCA: A sine cosine algorithm for solving optimization problems. Knowl.-Based Syst..

[52] Gaing Z.L. (2004). A particle swarm optimization approach for optimum design of PID controller in AVR system. IEEE Trans. Energy Convers..

[53] Wang R.-B., Hu R.-B., Geng F.-D., Xu L., Chu S.-C., Pan J.-S., Meng Z.-Y., Mirjalili S. (2025). The Animated Oat Optimization Algorithm: A nature-inspired metaheuristic for engineering optimization and a case study on Wireless Sensor Networks. Knowl.-Based Syst..

[54] Ćalasan M., Micev M., Radulović M., Zobaa A.F., Hasanien H.M., Abdel Aleem S.H. (2021). Optimal PID controllers for AVR system considering excitation voltage limitations using hybrid equilibrium optimizer. Machines.

[55] Izci D., Ekinci S., Mirjalili S. (2023). Optimal PID plus second-order derivative controller design for AVR system using a modified Runge Kutta optimizer and Bode’s ideal reference model. Int. J. Dyn. Control.

[56] Köse E. (2020). Optimal control of AVR system with tree seed algorithm-based PID controller. IEEE Access.

[57] Gozde H., Taplamacioglu M.C. (2011). Comparative performance analysis of artificial bee colony algorithm for automatic voltage regulator (AVR) system. J. Frankl. Inst..

[58] Micev M., Ćalasan M., Ali Z.M., Hasanien H.M., Aleem S.H.A. (2021). Optimal design of automatic voltage regulation controller using hybrid simulated annealing–Manta ray foraging optimization algorithm. Ain Shams Eng. J..

[59] Ekinci S., Can Ö., Izci D. (2025). Controller design for automatic voltage regulator system using modified opposition-based weighted mean of vectors algorithm. Int. J. Model. Simul..

